# Dual-Energy Micro-CT Functional Imaging of Primary Lung Cancer in Mice Using Gold and Iodine Nanoparticle Contrast Agents: A Validation Study

**DOI:** 10.1371/journal.pone.0088129

**Published:** 2014-02-10

**Authors:** Jeffrey R. Ashton, Darin P. Clark, Everett J. Moding, Ketan Ghaghada, David G. Kirsch, Jennifer L. West, Cristian T. Badea

**Affiliations:** 1 Center for In Vivo Microscopy, Duke University Medical Center, Durham, North Carolina, United States of America; 2 Department of Pharmacology and Cancer Biology, Duke University Medical Center, Durham, North Carolina, United States of America; 3 The Edward B. Singleton Department of Pediatric Radiology, Texas Children’s Hospital, Houston, Texas, United States of America; 4 Department of Radiation Oncology, Duke University Medical Center, Durham, North Carolina, United States of America; 5 Department of Biomedical Engineering, Duke University, Durham, North Carolina, United States of America; Cincinnati Children’s Hospital Medical Center, United States of America

## Abstract

**Purpose:**

To provide additional functional information for tumor characterization, we investigated the use of dual-energy computed tomography for imaging murine lung tumors. Tumor blood volume and vascular permeability were quantified using gold and iodine nanoparticles. This approach was compared with a single contrast agent/single-energy CT method. *Ex vivo* validation studies were performed to demonstrate the accuracy of *in vivo* contrast agent quantification by CT.

**Methods:**

Primary lung tumors were generated in *LSL-Kras^G12D^; p53^FL/FL^* mice. Gold nanoparticles were injected, followed by iodine nanoparticles two days later. The gold accumulated in tumors, while the iodine provided intravascular contrast. Three dual-energy CT scans were performed–two for the single contrast agent method and one for the dual contrast agent method. Gold and iodine concentrations in each scan were calculated using a dual-energy decomposition. For each method, the tumor fractional blood volume was calculated based on iodine concentration, and tumor vascular permeability was estimated based on accumulated gold concentration. For validation, the CT-derived measurements were compared with histology and inductively-coupled plasma optical emission spectroscopy measurements of gold concentrations in tissues.

**Results:**

Dual-energy CT enabled *in vivo* separation of gold and iodine contrast agents and showed uptake of gold nanoparticles in the spleen, liver, and tumors. The tumor fractional blood volume measurements determined from the two imaging methods were in agreement, and a high correlation (R^2^ = 0.81) was found between measured fractional blood volume and histology-derived microvascular density. Vascular permeability measurements obtained from the two imaging methods agreed well with *ex vivo* measurements.

**Conclusions:**

Dual-energy CT using two types of nanoparticles is equivalent to the single nanoparticle method, but allows for measurement of fractional blood volume and permeability with a single scan. As confirmed by *ex vivo* methods, CT-derived nanoparticle concentrations are accurate. This method could play an important role in lung tumor characterization by CT.

## Introduction

Lung cancer remains the leading cause of cancer death worldwide, and the number of deaths attributed to lung cancer is expected to increase 50% by 2020 [1]. Computed tomography (CT) is the standard imaging test for the assessment of patients with suspected lung cancer. Unfortunately, malignant and benign lung nodules often show similar radiographic features in CT [2], so malignancy cannot always be properly identified and treated. In the National Lung Cancer Screening Trial (NLCST), CT screening in high-risk patients reduced lung cancer-specific mortality [3], and the United States Preventative Services Task Force has recently recommended routine CT lung cancer screening for high risk patients; however, this screening inevitably leads to the detection of small pulmonary nodules (<1 cm) that are not well-characterized by currently available imaging modalities (PET, CT, or MRI) [4]. The NLCST proved that most of these nodules are not clinically significant. Consequently, there is a need to extend the information provided by CT imaging for nodule characterization–both for characterizing known tumors and for differentiating newly-detected malignant tumors from benign nodules.

One potential method for additional tumor characterization is to study lung tumor vasculature. Angiogenesis is a key factor in cancer growth and metastasis. In particular, angiogenesis promotes tumor growth by supplying tumors with required nutrients and provides a conduit for metastatic spread. Tumor vasculature formed by rapid angiogenesis tends to be less well-organized and more permeable than normal vasculature [5]. The density of tumor vasculature, which is often correlated with the extent of angiogenesis, is related to tumor aggressiveness and can correlate with survival [6]. There is also evidence that the extent of vascularity and vascular permeability differs between benign and malignant pulmonary nodules [7]; however, more comprehensive studies, which are best performed at the preclinical level, are needed to elucidate the importance of these vascular biomarkers in lung cancer.

Using preclinical studies in mice, we have previously shown that indolent and aggressive lung tumors can be differentiated using single energy micro-CT and an iodine-containing liposomal (Lip-I) contrast agent [8]. Increased accumulation of nanoparticles (due to increased vascular permeability) was demonstrated in more aggressive tumors compared to indolent ones, while vascular density was similar in the two tumor types. The single contrast agent experiments required two CT scans separated by several days to allow for complete blood clearance of the contrast agent in order to quantify and differentiate vascular signal from tumor accumulation signal. The delay between early and delayed-phase imaging may not be needed if we adopt a more elegant solution involving two distinct types of nanoparticles and CT imaging methods such as spectral or dual-energy (DE) CT.

DE imaging is an advanced technique that has recently risen to the forefront of CT technology [9]. Absorption of x-rays by contrast agents is strongly dependent on both the contrast agent’s atomic weight and the energy of the incident x-ray. Thus, two materials of different atomic weight can be differentiated from one another based on their unique attenuation coefficients at two different x-ray energies. DE-CT allows selective visualization and quantification of multiple contrast agents in a single scan. DE-CT has made significant progress in clinical cancer imaging. For lung tumors, it has been shown that iodine enhancement in clinical DE-CT scans can be correlated with the SUV_max_ of ^18^FDG- PET [10], [11], showing that DE-CT has the potential to extend CT imaging beyond purely anatomical imaging to functional and molecular imaging. While DE-CT shows high promise in the clinic for cancer imaging, it has not been largely adopted in the preclinical domain due to the challenges associated with higher spatial resolution, temporal resolution, and noise levels in micro-CT. We, however, have been able address some of these challenges and have shown that DE micro-CT using nanoparticles as contrast agents can play an important role in preclinical imaging for cardiac [12], lung [13], and cancer applications [14], [15].

Nanoparticle contrast agents are essential to preclinical CT studies because conventional contrast agents are cleared from the bloodstream too quickly for effective imaging. Most preclinical animal models, especially mice, are able to renally clear low molecular weight clinical contrast agents from their blood within seconds (normal blood half-life in humans is 2–3 hours [16]), due to their extremely high cardiac output. Nanoparticles used as CT contrast agents typically have a long (>2 hour) half-life and also tend to accumulate in tumors due to the enhanced permeability and retention (EPR) effect [17]. The utility of these agents in preclinical CT imaging has been well established [14], [18]–[24].

We have recently developed a DE micro-CT method to separate gold and iodine-based nanoparticles for vascular imaging in soft-tissue sarcomas [14]. In this method, gold nanoparticles are injected and allowed to accumulate in tumor tissue for two days. Liposomal iodine is then injected and immediately followed by a DE-CT scan while the iodine remains intravascular. Vascular permeability (rate of tumor nanoparticle uptake) is calculated using the measured gold concentration in the tissues, while blood volume is calculated from tissue iodine concentrations. The present work aims to apply the DE micro-CT method to the challenging task of imaging lung tumors for quantification of tumor blood volume and vascular permeability. In this study, we show that the two contrast agent (two-material) method, which requires only a single CT scan, compares favorably with our previously published single contrast agent (single-material) method, which required two CT scans spaced several days apart [8]. Importantly, we also aim to validate our *in vivo* dual-energy results with *ex vivo* gold standard measurements. To the best of our knowledge, there have been no published clinical or preclinical DE-CT studies with rigorous validation of calculated *in vivo* material concentrations. DE measurements are usually calibrated using *in vitro* phantoms, which approximate, but do not fully represent, *in vivo* conditions. Therefore, there is the potential for significant error in DE measurements when relying only on in vitro phantom calibrations. In this manuscript, we undertake *in vivo* validation, which is critical to further develop DE-CT for preclinical models.

## Materials and Methods

### Ethics Statement

All animals were handled in accordance with good animal practice as defined by the relevant national and/or local animal welfare bodies, and all animal work was approved by the Institutional Animal Care and Use Committee (IACUC protocol A283-11-11) of Duke University Medical Center. The Duke University Medical Center animal management program is accredited by the American Association for Assessment and Accreditation of Laboratory Animal Care (AAALAC) and meets National Institutes of Health standards as set forth in the “Guide for the Care and Use of Laboratory Animals” (DHHS Publication No. (NIH) 85-23, Revised 1985). The institution also accepts as mandatory the PHS “Policy on Humane Care and Use of Laboratory Animals by Awardee Institutions” and “NIH Principles for the Utilization and Care of Vertebrate Animals Used in Testing, Research and Training.”.

### Liposomal Iodine Fabrication

Liposomal iodine was produced as described previously [25]. A lipid mixture (200 mmol/L) consisting of 1,2-dipalmitoyl-sn-glycero-3-phosphocholine (DPPC), cholesterol, and 1,2-distearoyl-sn-glycero-3-phosphoethanolamine-N-[methoxy(polyethylene glycol)-2000] (DSPE-MPEG 2000) in a 55∶40:5 molar ratio was dissolved in ethanol and hydrated with a concentrated iodixanol solution (550 mg I/mL). The resulting lipid solution was sequentially extruded on a Lipex Thermoline extruder (Northern Lipids, Vancouver, British Columbia, Canada) to size the liposomes to ∼100 nm. The liposome solution was diafiltered using a MicroKros® module (Spectrum Laboratories, Milpitas, CA) to remove un-encapsulated iodixanol.

### Liposomal Iodine Characterization

The size distribution of liposomes in the final formulation was determined by dynamic light scattering (DLS) using a Malvern Zetasizer Nanoseries (Malvern Instruments, Worcestershire, UK) at 25°C. Transmission electron microscopy (TEM) was performed for additional size analysis and contrast agent characterization. Liposomes were spot-dried on a carbon film grid and imaged using an FEI Tecnai G^2^ Twin TEM (FEI, Hillsboro, OR) at an operating voltage of 120 mV. Particle diameter was measured for 200 liposomes using ImageJ (http://rsbweb.nih.gov/ij/, NIH). The iodine concentration in the final liposomal solution was quantified by measuring UV absorbance at 245 nm using a Cary 50 spectrophotometer (Varian Medical Systems, Palo Alto, CA). *In vitro* stability of the liposomes was verified by measuring release of iodixanol from the liposomes after incubation in phosphate-buffered saline (PBS) at 37°C for 72 hours. The concentration of iodixanol released following incubation was determined by dialyzing the liposomes against 500 mL PBS and measuring the UV absorbance of the dialysate in a quartz cuvette.

### Gold Nanoparticle Fabrication

Gold nanoparticles (AuNPs) were produced using the Frens method [26]. 500 mL of a 1 mM solution of HAuCl_4_ in ultrapure water was brought to boiling. A pre-warmed solution of 540 mg sodium citrate dissolved in 3 mL of water was rapidly injected into the gold solution and the resulting mixture was stirred vigorously. The color changed from yellow to colorless to dark red over the course of 30 seconds. The reaction was continued at boiling for 15 minutes, after which the reaction vessel was removed from the heat source and allowed to cool with continued stirring for 1 hour. After cooling, the nanoparticles were filtered using a 0.45-µm polyethersulfone filter. To passivate the produced nanoparticles, a large excess of 5 kDa thiol-terminated polyethylene glycol (PEG; Laysan Bio, Arab, AL) was added to the filtered nanoparticles, which were then rocked at room temperature for 4 hours. Unbound PEG molecules were removed and the particles concentrated using a 100 kDa centrifugal filter.

### Gold Nanoparticle Characterization

The size distribution and surface charge of the gold nanoparticles were characterized by DLS and zeta potential using a Malvern Zetasizer Nanoseries at 25°C. DLS size distribution was confirmed by TEM using an FEI Tecnai G^2^ Twin TEM operating at 200 mV. Particle diameter and aspect ratio were measured for 200 AuNPs using ImageJ. Absorbance spectra of AuNPs suspended in ultrapure water were collected from 400 to 650 nm using a UV-Vis spectrophotometer. Stability of the PEGylated nanoparticles against aggregation was confirmed by monitoring the UV-Vis spectrum for 6 hours after addition of physiological salt solutions of 0.9% NaCl or Dulbecco’s Modified Eagle Medium (DMEM) culture medium with 10% fetal bovine serum (FBS) to solutions of bare or PEGylated AuNPs. The final concentration of gold in the concentrated AuNP solution was determined by UV-Vis absorbance using the published extinction coefficient of 12 nm AuNPs at 450 nm [27] and was subsequently correlated with measurements from inductively-coupled plasma optical emission spectroscopy (ICP-OES), as described below.

### *In Vivo* Tumor Imaging

Lung tumors were generated by intranasal injection of adenovirus expressing Cre recombinase (Gene Transfer Vector Core, University of Iowa) into *LSL-Kras^G12D^; p53^FL/FL^* compound mutant mice as described previously [28]–[30]. Mice with primary lung tumors were used for the imaging study at 12 weeks post Adeno-Cre infection, at which point multiple aggressive lung adenocarcinomas (∼0.5–1.5 mm in diameter) were present within each mouse. All animals were imaged at 24–30 weeks of age. A total of five animals were used for the imaging study. Due to the longitudinal nature of this study, each mouse served as its own control.

**Figure 1:**
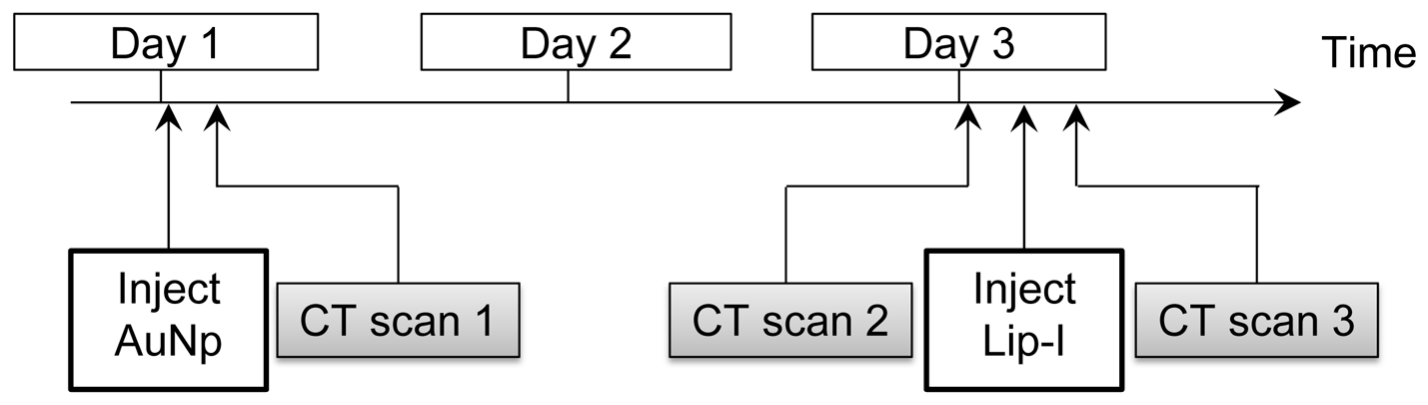
Flowchart for contrast injection and imaging time points. AuNPs were injected on day 1, followed immediately by CT scan 1. Two days later, CT scan 2 was done, followed immediately by Lip-I injection and CT scan 3. All scans were dual-energy micro-CT acquisitions.

Longitudinal DE micro-CT imaging was performed in all animals as shown in Figure 1. Three DE micro-CT scans were performed for each mouse in order to compare the single-material method (CT scans 1 and 2) with our novel two-material method (CT scan 3). We note that, unlike in our previous study [14], DE-micro-CT was performed even when using a single contrast agent, which allowed us to measure gold concentration without the need for a comparison pre-contrast scan. On day 1, the AuNP contrast agent was injected intravenously by tail vein at a volume dose of 0.32 mL/25 gm body weight and post-injection scans (CT scan 1) were immediately acquired. We then waited 48 hours to allow sufficient time for gold accumulation to occur in the tumors. After this delay (day 3) a second dual-energy CT scan (CT scan 2) was obtained. Immediately after the second scan, the liposomal iodine contrast agent was injected by tail vein at a volume dose of 0.3 mL/25 gm body weight and a third dual-energy CT scan (CT scan 3) was performed. Following the third scan, the mice were euthanized and tissues were harvested for validation studies, as described below.

### Dual-Energy Micro-CT System

A custom-built dual-source micro-CT imaging system was used for the study [31]. The animals were scanned while free breathing under anesthesia using 2–3% isoflurane delivered by nose-cone. Body temperature was maintained with heat lamps, a rectal probe, and a feedback controller (Digi-Sense®, Cole Parmer, Chicago, IL). Prospective respiratory gating was used to minimize the effects of animal respiratory motion during scans [32]. A pneumatic pillow positioned on the animals’ thorax connected to a pressure transducer was used to monitor breathing. A LabVIEW (National Instruments, Austin, TX) application was used to monitor the respiratory signal and trigger the x-ray tubes at end expiration by computing a fixed time delay from the peak respiratory signal. Acquisitions were performed for both imaging chains at each angle of rotation with a 10 msec delay between the two x-ray tube exposures to minimize cross-scatter. Since this is a very short delay relative to the length of the flat end expiratory phase, it does not affect the performance of respiratory gating. A total of 360 views were acquired for each imaging chain over the 360° rotation. Each prospectively-gated scan took approximately 7 minutes to complete. The scanning parameters for the dual-energy scans were: 80 kVp, 160 mA, 10 ms/exposure for the first imaging chain and 40 kVp, 250 mA, 16 ms/exposure for the second imaging chain. The total radiation dose associated with the three CT scans was 0.39 Gy.

### Post-processing and Dual-Energy Decomposition

**Figure 2:**
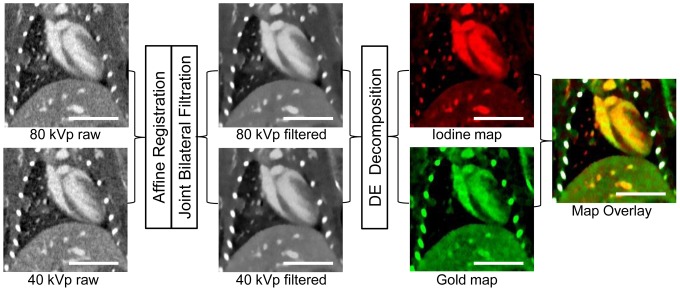
Dual-energy decomposition process diagram. Raw 40 kVp and 80 kVP datasets were acquired, and then they underwent affine registration and joint bilateral filtration to produce filtered data sets. Dual-energy decomposition was performed on these filtered images, which resulted in two independent images (maps) representing the iodine and gold concentration in each voxel. After obtaining the two maps, the images were overlaid and the bones were segmented out (colored white) to form the final concentration map overlay. These images were taken from CT scan 3, in which both iodine and gold were present in the blood stream. The scale bar represents 1 cm in all images. The 40 and 80 kVp images are windowed from −300 to 1200 HU, the iodine maps are windowed from 0.25 to 15 mg/mL, and gold maps are windowed from 0.25 to 6 mg/mL.

DE micro-CT data processing followed the flowchart in Figure 2. Raw 40 kVp and 80 kVp datasets were reconstructed using the Feldkamp algorithm [33] in a matrix of 512×512×512 at 88-µm isotropic voxel size. Affine registration was performed to improve registration between corresponding 40 and 80 kVp reconstructed volumes using ANTs, an open-source, ITK-based registration toolkit (Advanced Normalization Tools, http://picsl.upenn.edu/ANTS/; svn 1409; [34], [35]). To improve the decomposition, each dataset was denoised using joint bilateral filtration (BF). Joint BF is a spectral extension of the well-characterized edge-preserving smoothing filter that considers the distribution of neighboring voxels in both space and intensity. Joint BF was implemented using MATLAB (MathWorks, Natick MA), and generally completed within 15 to 20 minutes per set of energies. Details of the application of joint BF to murine micro-CT data [36] and a quantitative evaluation of the application of BF to DE micro-CT [13] have been described previously.

DE decomposition of gold and iodine was performed after registration and filtration, using corresponding 80 and 40 kVp-filtered data, as described previously [14], [37]. For each voxel, the decomposition involved solving a system of two equations with two unknowns, C_I_ and C_Au_, which represent the concentrations of iodine and gold, respectively, within the given voxel. The measured CT attenuation value in each voxel represents a linear combination of the unknown gold and iodine concentrations in mg/mL multiplied by the coefficients of the CT sensitivity matrix as shown in the following equations:
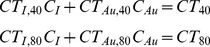



Here, C_I_ and C_Au_ are the unknown concentrations of iodine and gold, respectively, in a given voxel, CT_40_ and CT_80_ are the measured attenuations in the voxel at 40 and 80 kVp, and CT_I,40_, CT_I,80_, CT_Au,40_, and CT_Au,80_ are empirically-determined coefficients of the constant sensitivity matrix for iodine and gold at 40 and 80 kVp in measured attenuation (HU units) per contrast agent concentration (mg/mL). The unknown concentrations of iodine and gold in each voxel were determined by inverting this sensitivity matrix and finding the least squares solution of the following system of equations in MATLAB:
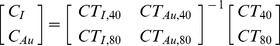



Scanning energies for optimal gold and iodine decomposition were chosen following the results of our previous simulations and *in vitro* studies [37]. These studies showed that the maximum spectral difference between gold and iodine for polychromatic x-ray sources occurred at operating voltages of 40 and 80 kVp. Values for the coefficients of the sensitivity matrix at each energy (CT_I,40_, CT_I,80_, CT_Au,40_, and CT_Au,80_) were determined empirically using a calibration phantom as described previously [14]. For the phantom calibration, the CT attenuation of vials of known gold and iodine concentration were measured at 40 and 80 kVp, and the coefficients for the sensitivity matrix were determined by fitting the known gold and iodine concentrations to the measured CT attenuation using a linear least squares regression at each energy level. The derived values for CT_I,40_, CT_I,80_, CT_Au,40_, and CT_Au,80_ used in this study were 28.7, 42.6, 88.5, and 58.8 HU/mg/mL, respectively. Following decomposition, voxels with negative concentrations of both materials were set to zero. Voxels with a negative concentration of one material and a positive concentration of the other material were projected onto the subspace of positive concentration. *In vitro* validation of this decomposition method using both digital and physical phantoms has been shown in our previous work [14]. These validation studies demonstrated the ability of this decomposition method to accurately measure gold and iodine concentrations both when they are independent from one another and when they are present in the same voxel.

### Image Analysis

CT images were analyzed using Avizo (FEI Visualization Sciences Group, Burlington, MA). Regions corresponding to the blood (ventricular lumen and large vessels) and spleen were automatically segmented using the 80 kVp data set, while regions corresponding to the liver and kidneys were semi-automatically segmented using the same data set. Each segmented region included the entire organ, but excluded large blood vessels within the organ. Tumors were segmented semi-automatically using the gold map from the dual-energy decomposition. Rough regions were manually drawn outside the borders of the tumor, and those regions were thresholded to select only those voxels that contained a gold concentration between 0.25 mg/mL and 3 mg/mL. These threshold values were chosen in order to exclude normal lung parenchyma, large blood vessels, and bone from the segmented tumors. A total of 27 lung tumors in the 5 mice were identified and segmented in each of the 3 DE micro-CT scans.

Following segmentation, gold and iodine concentrations were measured in each segmented region of interest by calculating the average value of the gold and iodine maps over the entire region of interest. Because both nanoparticles are long half-life contrast agents, they remain almost entirely within the vasculature immediately after injection. The fractional blood volume (FBV) of each organ can, therefore, be estimated by measuring the gold or iodine concentration within the organ immediately after nanoparticle injection. We calculated FBV on day 1 using the AuNPs and again on day 3 using the Lip-I. FBV was calculated by the following equations:
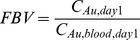


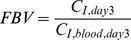
where C_Au,day1_ is the average gold concentration in a tissue immediately after gold injection on day 1, C_Au,blood,day1_ is the average gold concentration in the segmented blood on day 1, C_I,day3_ is the average iodine concentration in a tissue immediately after Lip-I injection, and C_I,blood,day3_ is the average iodine concentration measured in the segmented blood on day 3. FBV was calculated for each segmented tumor, spleen, liver, and kidney at both time points. This calculated FBV is an estimate of the vascular density within the tissue, and was correlated with microvascular density calculated by analyzing images of histological tissue sections, as described below.

After determining the FBV in each tissue, the concentration of gold accumulated within each tissue was calculated. Because of its long blood half-life, over half of the injected contrast agent remained in the blood stream on day 3. Therefore, the tissue gold concentration measured in the CT gold map includes both gold that extravasated into the tissue and gold that remains intravascular within the tissue. The FBV calculated above can be used to subtract the intravascular gold concentration according to the following equations:
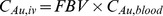


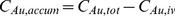
where C_Au,accum_ is the accumulated (extravascular) gold concentration in a tissue, C_Au,tot_ is the total gold concentration in a tissue calculated from the CT gold map on day 3, C_Au,iv_ is the concentration of gold within a tissue that remains intravascular, and C_Au,blood_ is the concentration of gold in the bulk blood calculated from the CT gold map on day 3. These equations were used to calculate the accumulated gold within each tumor and organ. For the single contrast agent method, the FBV from CT scan 1 and the gold concentrations from CT scan 2 were used in these calculations. For the two contrast agent method, the FBV and gold concentrations were both from CT scan 3. For validation, these accumulated gold concentrations were compared to concentrations of gold in each organ measured by ICP-OES.

### Validation Studies

#### Tissue processing

Samples of blood, lung tumors, and other organs (kidney, liver, spleen) were extracted from all mice for analysis. Blood was drawn from the mice following the first CT scan from the facial vein. A terminal blood draw and organ harvesting were performed following the third CT scan. Each mouse was put under deep anesthesia by an intraperitoneal injection of pentobarbital. Anesthesia was verified by toe pinch. The abdominal cavity was opened and blood was drawn slowly from the inferior vena cava while the heart was still beating. 0.5–1.0 mL of blood was collected from each mouse, after which the inferior vena cava and aorta were severed and the remaining blood was allowed to drain into the abdominal cavity. The trachea was then cannulated with a 20-gauge needle, through which a 1∶1 mixture of cryoembedding medium (OCT) and 30% sucrose were injected into the lungs to fill the airspaces for later tissue sectioning. Following inflation with embedding medium, the lungs were removed from the mouse and either immediately dissected for lung tumors or embedded in OCT and frozen on dry ice. Lung tumors extracted from the dissected lungs were soaked in PBS for 5 minutes to rinse away residual blood and then frozen at –80°C for ICP-OES analysis. Following lung extraction, the liver, spleen, and kidneys of each mouse were harvested and cut in half. The first half of each organ was immediately embedded in OCT on dry ice for sectioning. The second half was soaked in PBS for 5 minutes and frozen at −80°C for ICP-OES analysis. All tissues were kept frozen at –80°C until ready for further processing.

#### Histology

8-µm frozen tumor sections were immunostained for the endothelial cell marker CD31. Prior to staining, sections were fixed with 4% paraformaldehyde, rinsed with PBS, and blocked with 10% FBS in PBS for one hour. The sections were then incubated with the primary antibody (rat anti-mouse CD31, BD Pharmingen) diluted 1∶250 in blocking buffer for 2 hours at room temperature. The slides were rinsed three times with PBS for 10 minutes to remove unbound primary antibody, after which the secondary antibody (Alexa Fluor488-conjugated donkey anti-rat IgG, Invitrogen) diluted 1∶500 in blocking buffer was added and incubated for 1 hour in the dark. Nuclei were counterstained with DAPI. A few 8-µm sections were also used for hematoxylin and eosin (H&E) staining. For H&E staining, samples were stained with hematoxylin, rinsed with water and ethanol, stained with eosin Y, then rinsed with ethanol and xylene and mounted for microscopy.

Immunostained sections were imaged using a Zeiss Axiovert 135 inverted microscope (Carl Zeiss Inc., Thornwood, NY) and a Cytoviva darkfield condenser and dual mode fluorescence module (CytoViva, Inc., Auburn, AL) fitted with a triple bandpass emission filter. All images were acquired at 200× magnification. Images were taken of each tumor using both fluorescence and darkfield modes. With darkfield imaging, indirect sample illumination enables image production from light scattered by the samples. Gold nanoparticles are readily detected due to their increased scattering properties relative to the surrounding tissue. At least three images of each tumor were acquired when possible, but a few tumors were too small to obtain more than a single image.

Immunofluorescence images were analyzed to calculate tumor microvascular density. The green channel (corresponding to the CD31 stain) of each image was isolated and automatically thresholded using ImageJ. Microvascular density for each image was determined by calculating the percentage of each field of view that was positive for CD31 staining after thresholding and averaging that percentage over multiple images of the same tumor.

Combined darkfield and fluorescent images were produced by first thresholding the darkfield images so that only the bright gold nanoparticles remained, then overlaying the resulting gold nanoparticle image with the fluorescence image. This allowed visualization of the spatial relationship between the gold nanoparticles and the vasculature within the tumors.

#### Quantification of gold content within tissues

Blood samples, extracted tumors and organs (liver, spleen, kidneys), and samples of the AuNPs were prepared for analysis of gold concentration by ICP-OES. Samples were weighed, frozen, and then lyophilized. Dry samples were digested in trace metals grade aqua regia for 72 hours. The samples were then filtered through a 0.45-µm filter and diluted with 2% nitric acid to a final gold concentration of approximately 2 ppm (based on the estimated gold concentrations from CT imaging). For lung tumor analysis, all tumors from a single mouse were digested as a single combined sample to determine the average tumor gold concentration for each mouse. Standard solutions of known gold concentrations (8 concentrations ranging from 0.1–10 ppm) were prepared in 2% aqua regia using certified reference material Gold Standard for ICP (Fluka Analytical, Buchs, Switzerland). The standards and digested samples were analyzed by ICP-OES (Teledyne Leeman Laboratories, Hudson, NH).

#### Blood iodine quantification

Because organically-bound iodine is not stable during acid digestion, we instead used the optical absorbance of the iodixanol molecule to quantify iodine concentrations in the blood samples. Blood samples were diluted 1∶2,000 in 10 mM sodium dodecyl sulfate (SDS) to lyse the liposomes then centrifuged at 5,000 g for 10 minutes to remove cellular debris. The supernatant was passed through a 3 kDa centrifugal filter and the absorbance of the resulting filtrate was measured at 245 nm. Blood (without liposomes) processed in the same manner was used as a blank for absorbance readings. This absorbance was compared to a standard curve of known iodixanol concentrations in 10 mM SDS.

### Statistics

Fractional blood volume on day 1 and day 3 were compared using a paired t-test. Accumulated gold concentrations from the two CT methods were compared to each other and to ICP-OES results using a 1-way ANOVA. Blood concentrations by CT were compared to ICP-OES/UV-Vis results using a paired t-test. Fractional blood volume and accumulated gold concentration for each of three tumor sizes were compared using a 1-way ANOVA followed by a post-hoc Tukey test for multiple comparisons. All statistical tests were considered statistically significant at a p<0.05 level. All data are plotted as mean ± standard deviation.

## Results

### Gold Nanoparticle Characterization

**Figure 3:**
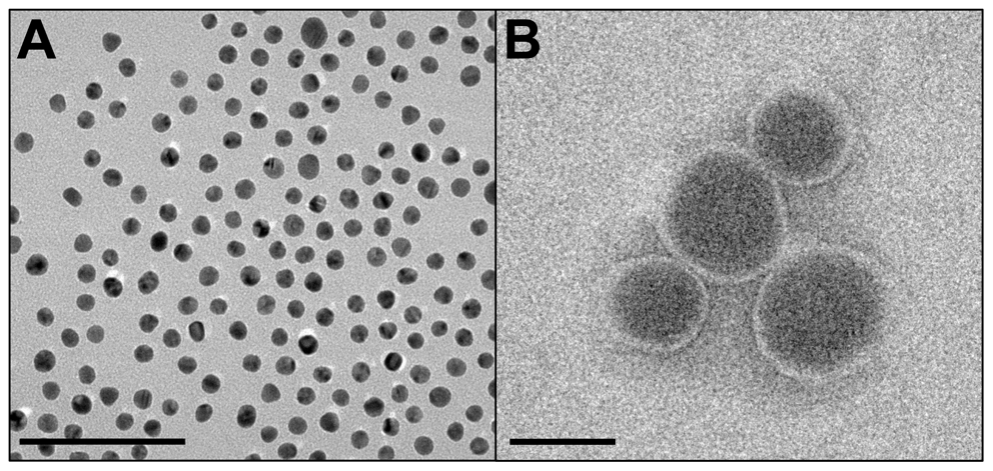
TEM of nanoparticles. PEGylated gold nanoparticles are shown in (A) and iodine-containing liposomes in (B). The scale bar represents 100 nm for both panels.

The absorbance spectrum of the bare gold nanoparticles (Figure S1) showed a peak at 518 nm, while the PEGylated nanoparticles had a peak absorbance at 521 nm, a shift consistent with successful conjugation of PEG to the nanoparticle surface. DLS showed a mean hydrodynamic diameter of 11.6±1.4 nm with an average polydispersity index (PDI) of 0.25 for the bare nanoparticles and a mean hydrodynamic diameter of 29.9±1.2 nm with a PDI of 0.27 for the PEGylated nanoparticles. Zeta potential measurements showed an average zeta potential of −37±2 mV for the bare nanoparticles and –2.6±0.4 mV for the PEGylated nanoparticles, showing displacement of negatively charged citrate ions by the PEG-thiol and good masking of the remaining surface charge by the long PEG molecules. Representative TEM images of the nanoparticles are shown in Figure 3. TEM of the AuNPs showed spherical gold nanoparticles with an average diameter of 12.1±1.9 nm and average aspect ratio of 1.1. Aggregation studies showed that PEGylated AuNPs were stable for >6 hours (no measureable change in peak absorbance) in both 0.9% NaCl and DMEM growth medium +10% FBS, indicating both stability to ion-induced particle aggregation and resistance to protein adsorption and resultant particle cross-linking and aggregation, while the bare nanoparticles aggregated completely within minutes of exposure to both physiological solutions. The final measured concentration of gold in the injected AuNP solution was 77.6 mg/mL by UV-Vis absorbance and 74.4 mg/mL by ICP-OES.

### Liposomal Iodine Characterization

The average liposome size measured by DLS was 126±20 nm and the PDI was 0.11. TEM of the Lip-I (see Figure 3) showed spherical liposomes with a dark interior and a light bilayer with an average diameter of 103±34 nm. Because the PEG coating is not visible on TEM, this size is a measure of the bare liposome. When fully hydrated, the PEG coating should increase the size of the liposomes by ∼10–20 nm, which is consistent with the DLS results. The final measured iodine concentration in the PEGylated liposomal-iodine formulation was 135.0 mg/mL by UV absorbance. Stability tests showed <1% leakage of encapsulated iodixanol after incubation at 37°C for 72 hours.

### CT Tissue Enhancement

**Figure 4:**
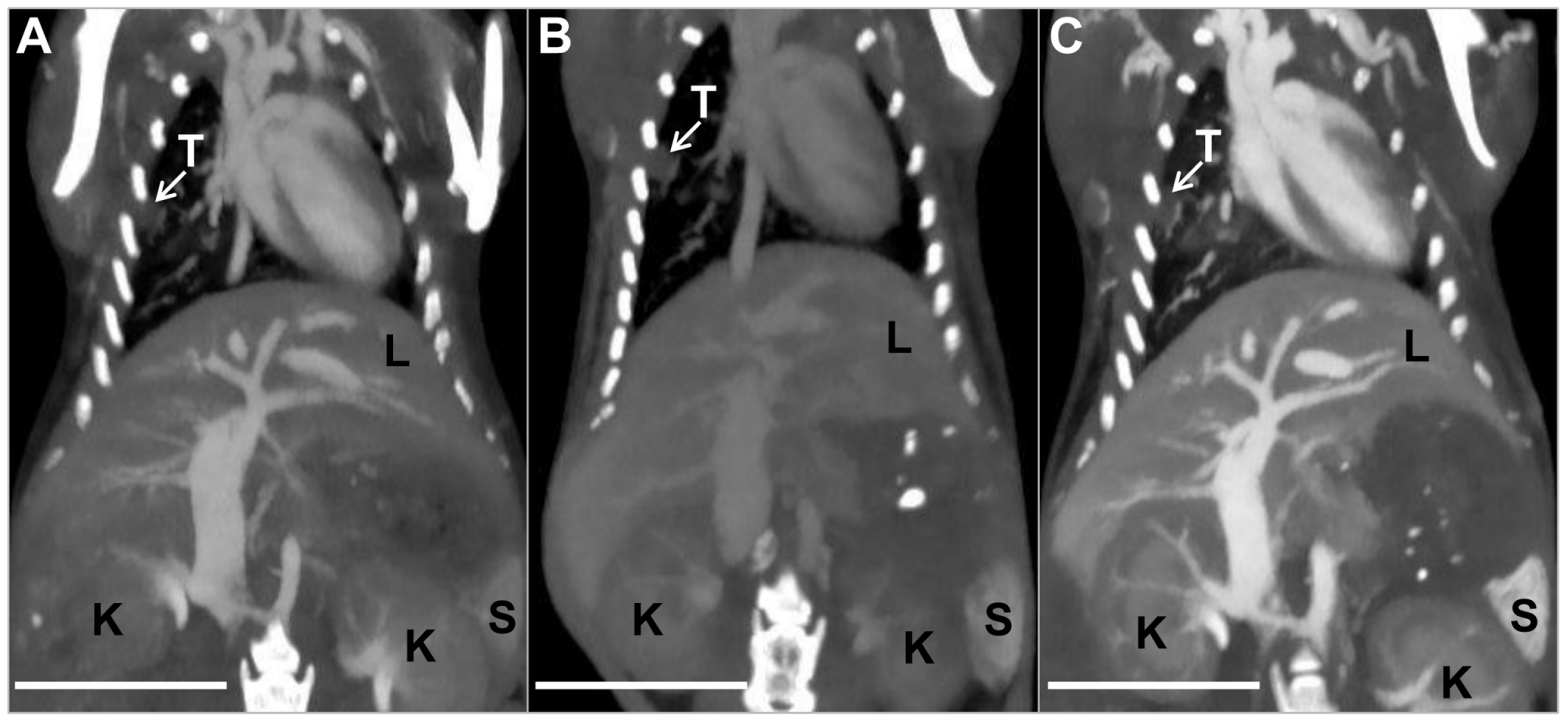
Thick slab maximum intensity projections of the 80 kVp CT datasets following bilateral filtration. CT scan 1 is shown in (A), CT scan 2 in (B) and CT scan 3 in (C). Tumor is marked “T,” liver is marked “L,” spleen is marked “S.” and kidneys are marked “K.” The images are all windowed between −300 and 1200 HU. The scale bar represents 1 cm in all panels.

**Figure 5:**
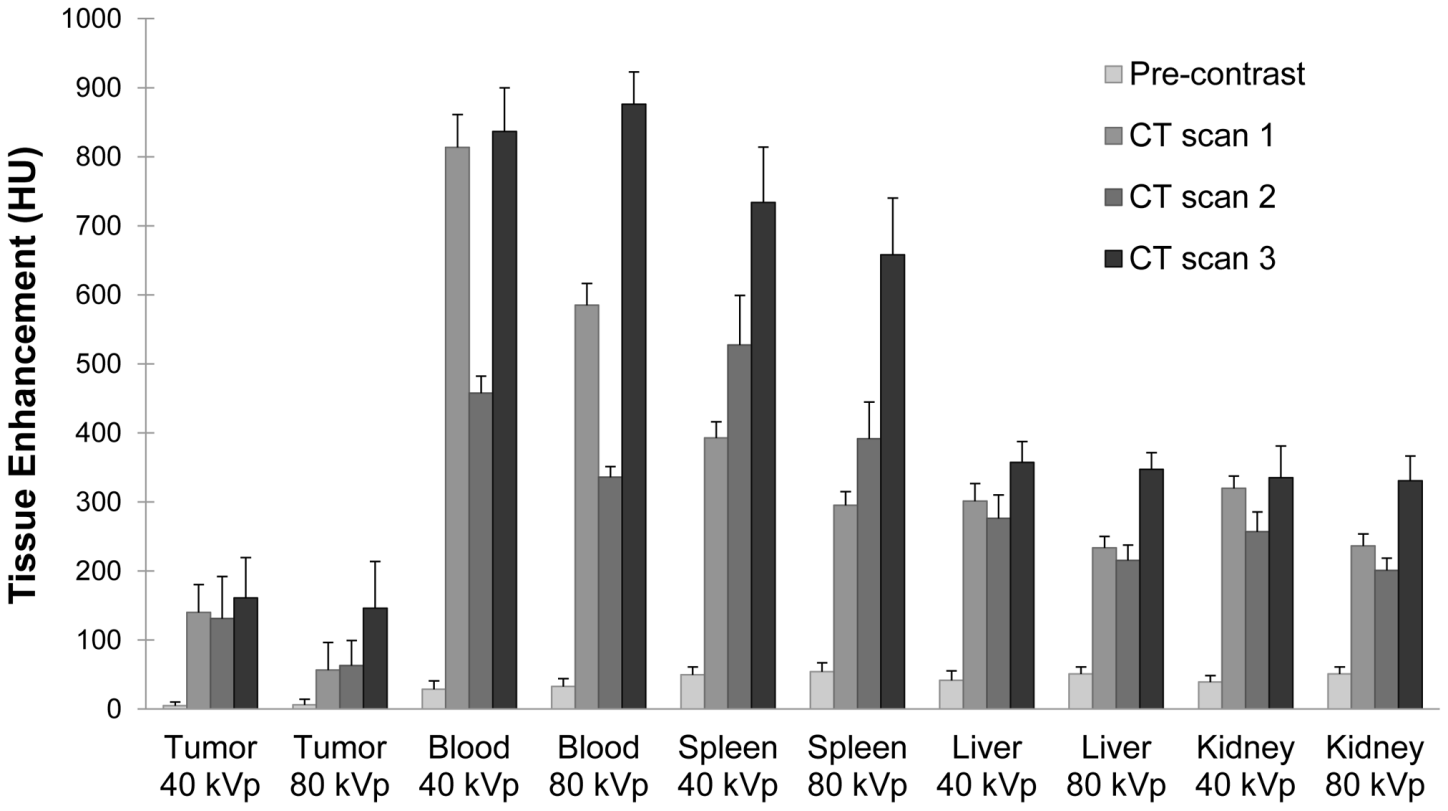
Average tissue enhancement. Tissue enhancement (average of 5 mice) is shown at 40 and 80 kVp for tumor, blood, spleen, liver, and kidneys in each of three CT scans. Pre-contrast values are the average tissue enhancement of the mice from pre-experiment monitoring scans.

Figure 4 shows CT images of a single mouse from each of the three scans, with labels marking a lung tumor, liver, spleen and kidneys. Figure 5 shows average enhancement within each organ at each of the time points, including pre-contrast values for each organ which were derived from earlier CT scans used for monitoring tumor size. After gold injection (CT scan 1), the blood showed high levels of contrast (∼800 HU at 40 kVp). The spleen, liver, kidneys, and tumors all showed moderate enhancement due to the gold within their vasculature. In each case, the 40 kVp images showed greater contrast than the 80 kVp images due to the higher attenuation of gold at 40 kVp relative to 80 kVp. After 48 hours, the average contrast in the blood dropped to 54% of its initial value, yielding a blood half-life of ∼54 hours (assuming exponential decay). While the contrast dropped in the blood, it remained approximately steady in the tumors, liver, and kidney from CT scan 1 to CT scan 2. Although the intravascular gold concentration was decreasing in these organs, gold was also accumulating extravascularly in the tissues, which made overall contrast levels remain the same. Tissue enhancement in the spleen increased from day 1 to day 3 due to significant gold accumulation within the spleen. Over this time period, enhancement within the other soft tissues (data not shown) decreased proportional to the decrease in blood levels. After iodine injection (CT scan 3), the contrast in the blood increased again. The 80 kVp data showed a significantly higher increase than the 40 kVp data, consistent with the relative attenuation of iodine at the two energy levels. Likewise, all of the organs measured showed a moderate increase in contrast at 80 kVp and a small increase in contrast at 40 kVp due to iodine within the vasculature of the organs.

**Figure 6:**
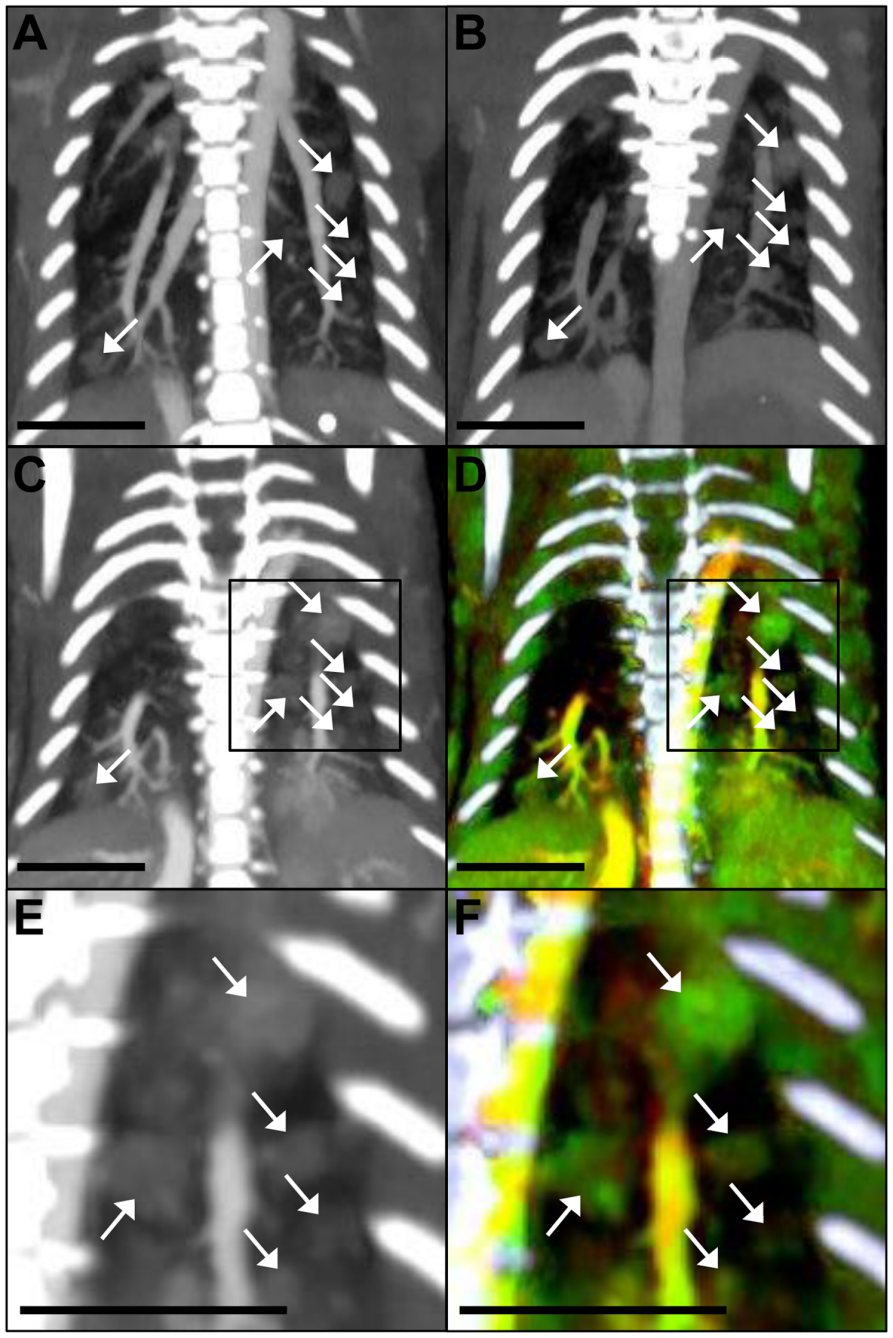
Thick slab maximum intensity projections of the lungs. Coronal CT images showing the filtered 80 kVp dataset from CT scan 1 (A), CT scan 2 (B), CT scan 3 (C), and the final overlay of the DE decomposition of CT scan 3 (D) with bones segmented out (bones in white). (E) is a zoomed-in image of the right side of CT scan 3, and (F) is a zoomed in image of the right side of the DE overlay of CT scan 3. The boxes in (C) and (D) show the regions that are enlarged in (E) and (F), respectively. 80 kVp images are windowed from –300 to 1200 HU. The overlays are windowed from 0.25 to 6 mg/mL gold, and 0.25 to 15 mg/mL iodine. The scale bar represents 0.5 cm in all images.

Figure 6 shows CT images focused on the lungs at each of the three time points in approximately the same slice for a single mouse, as well as the final overlay of the gold and iodine dual-energy decomposition maps (with bones segmented out and colored white) for the final time point. These images show multiple tumors (see arrows) in both lungs. The dual-energy decomposition shows both gold (green) and iodine (red) in the blood stream, which appears yellow in the overlay. The tumors are primarily green due to the accumulation of gold within the tumors. The liver at the bottom of the image shows up yellow-green, due to the combination of extravasated gold and intravascular gold and iodine within the liver. The soft tissue has a small amount of both gold and iodine due to intravascular contrast within the tissues. Around the ribs, there is a patchy distribution of green and red, primarily due to beam hardening artifacts from the dense ribs. The tumors enhanced significantly relative to the normal lung parenchyma at all three time points after contrast injection, which allowed for easy identification of the tumors, despite their small size (0.2–1.5 mm^3^). In several cases, the size and shape of individual tumors varied significantly between the different CT scans, but identification of these tumors at different time points was still possible based on their relative location within the lungs.

### Fractional Blood Volume

**Figure 7:**
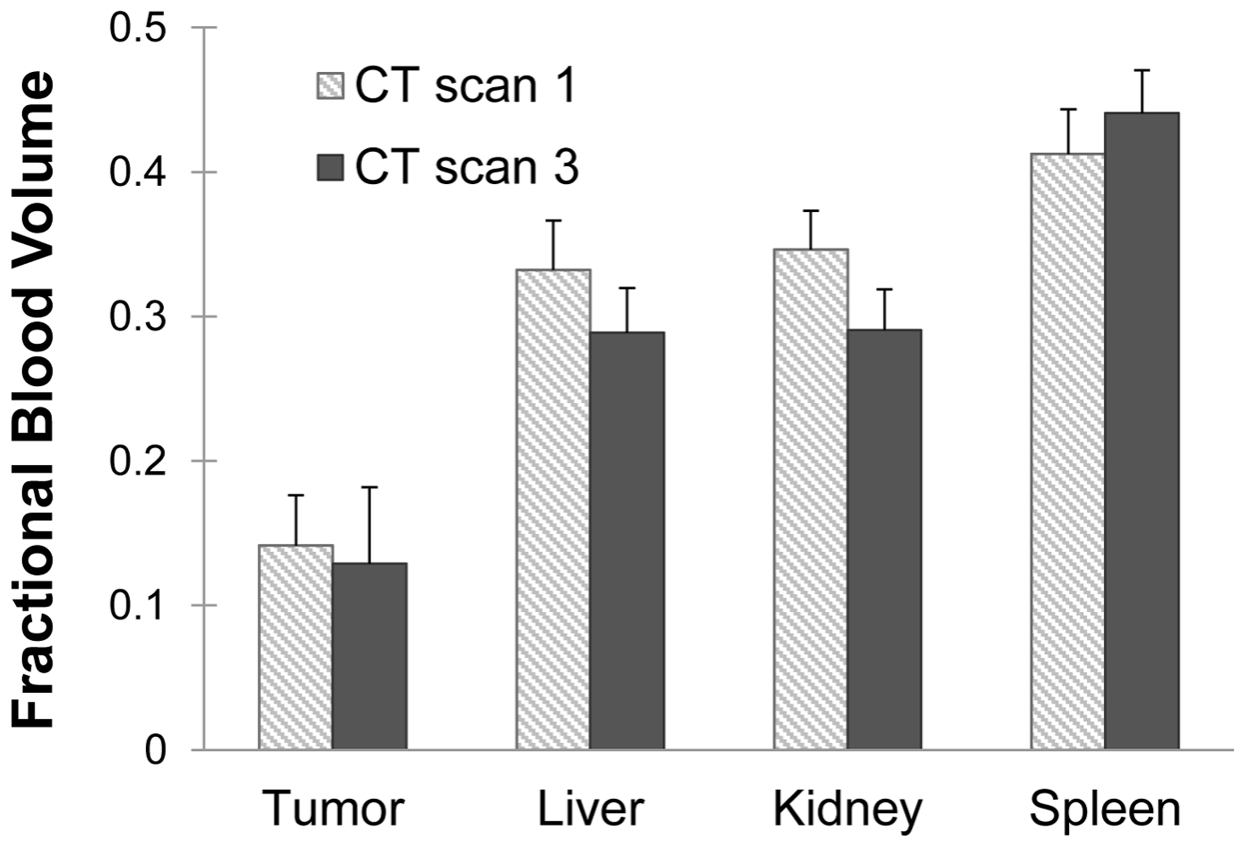
Fractional blood volume. Comparison of FBVs calculated from CT scan 1 (based on gold) and CT scan 3 (based on iodine) for each of the organs. None of the differences were found to be statistically significant (p<0.05).

Fractional blood volume for each tumor and organ was computed immediately after each contrast agent injection (using CT scan 1 and CT scan 3). The average FBV for each time point is shown in Figure 7. The average FBV for tumors after gold injection was 14%, while the average FBV after iodine injection was 13%, which was not a statistically significant difference. The actual FBV values for individual tumors ranged from 6% to 21%, showing some heterogeneity from one tumor to another. FBV calculation for the other organs also showed no statistically significant differences between the two measurements. The spleen had the highest fractional blood volume at 44% on day 3. The liver and kidney had approximately the same fractional blood volume at 29% on day 3.

### Validation Studies

#### Histology

**Figure 8:**
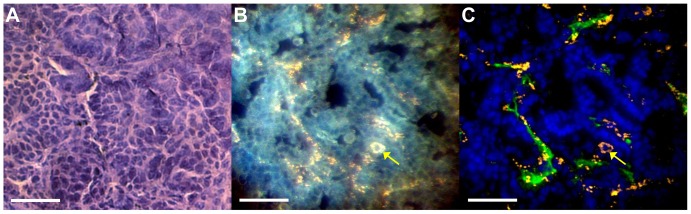
Lung tumor histology. (A) H&E staining of a lung tumor. (B) Darkfield microscopy of a different section of the same tumor. (C) The overlay of darkfield and fluorescence images with the nanoparticles from the darkfield image in orange, the CD31 stain in green and DAPI in blue. The yellow arrow denotes a cell (most likely a macrophage) with cytoplasm filled with nanoparticles. The scale bar represents 50 µm in all panels.

Figure 8 shows representative images of tumor sections with H&E staining, darkfield microscopy, and fluorescence microscopy. Tumor H&E staining showed dense regions of cells, with some areas forming acinar structures (including well-developed central blood vessels) and some areas showing highly irregular cells with large atypical nuclei and no clear organization. In general, the larger tumors had more areas with malignant characteristics (large nucleus to cytoplasm ratio, atypical cell morphology, loss of architecture, necrotic regions) and had more and larger blood vessels than smaller tumors, although there was a large amount of variability from tumor to tumor. Darkfield microscopy showed clusters of gold nanoparticles throughout the tumors, spleen, and liver. Most of the gold nanoparticles within the tumors were located near blood vessels (as shown in the fluorescence/darkfield overlays), but some nanoparticles extended farther into the interstitial space in most of the tumors. A large amount of gold nanoparticles were also found intracellularly in lung cells that were most likely alveolar macrophages (judging by cell morphology), as shown in Figure 8 with a yellow arrow. This was especially true in poorly-developed tumors that seemed to incorporate some normal lung alveoli within the tumor. There was also some gold in alveolar macrophages in the normal lung tissue. Darkfield images of cardiac muscle showed a very small amount of nanoparticle accumulation.

**Figure 9:**
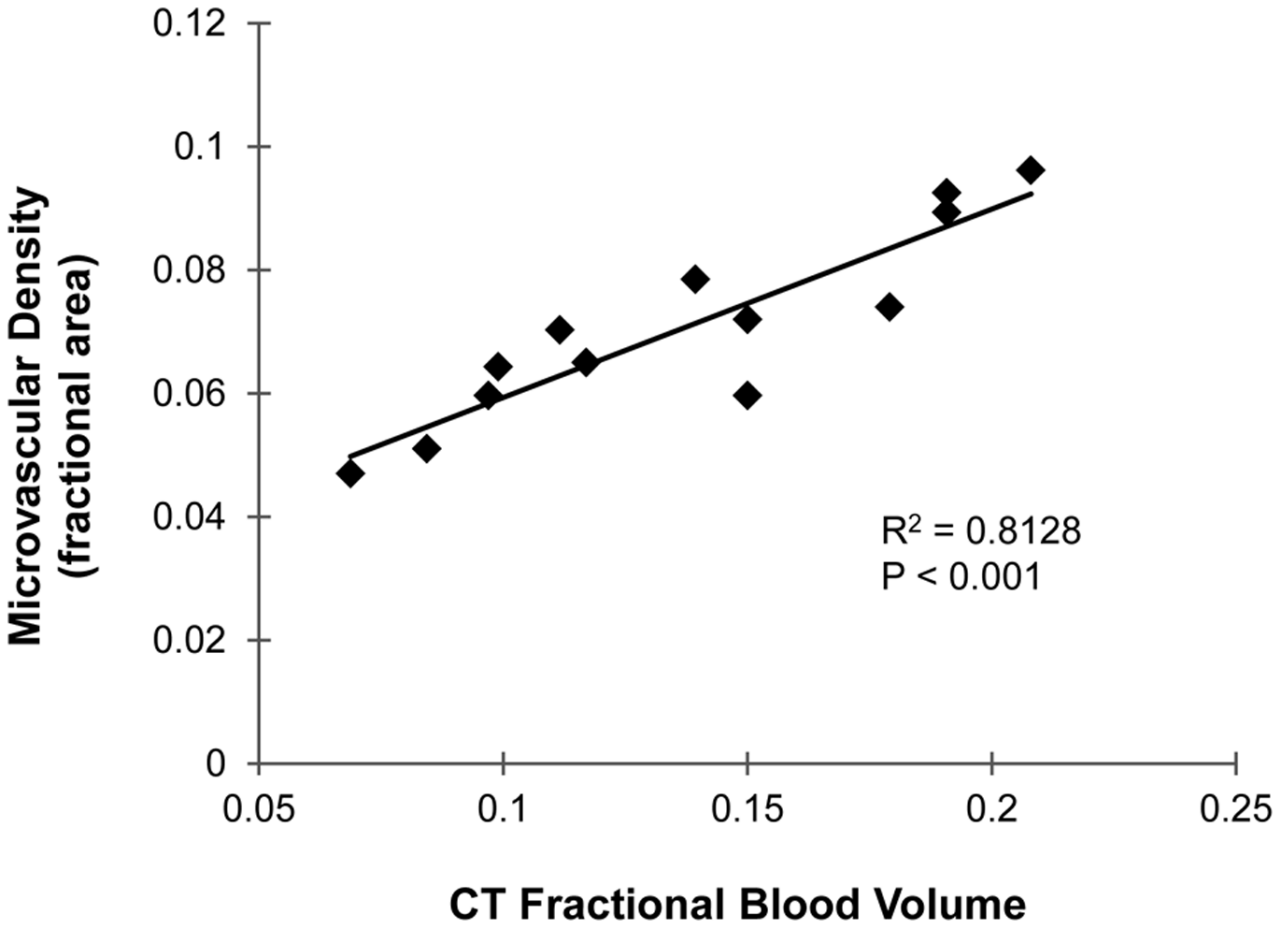
Microvascular density. Plot of microvascular density measured by CD31 immunostaining versus FBV measured by CT. Linear regression line is shown with the accompanying R^2^ value and p-value for the slope of the regression equation.

Fluorescence microscopy was also used to quantify the microvascular density (percent area staining positive for CD31) within the tumors as a comparison to the CT FBV. The average microvascular density measured by CD31 staining was 7%, compared to 14% for CT FBV. Figure 9 shows a plot of the CT-calculated FBV versus the histological microvascular density for each of 13 tumors which were successfully identified in both CT and histology images. Linear regression of the data gave an R^2^ value of 0.81, which shows strong correlation between the two methods. The p-value for the regression slope (testing the hypothesis that the slope does not equal zero) was <0.001, which shows that there is a statistically significant positive correlation between the CT and histology-derived measures of vascular density.

#### Gold and iodine quantification

**Figure 10:**
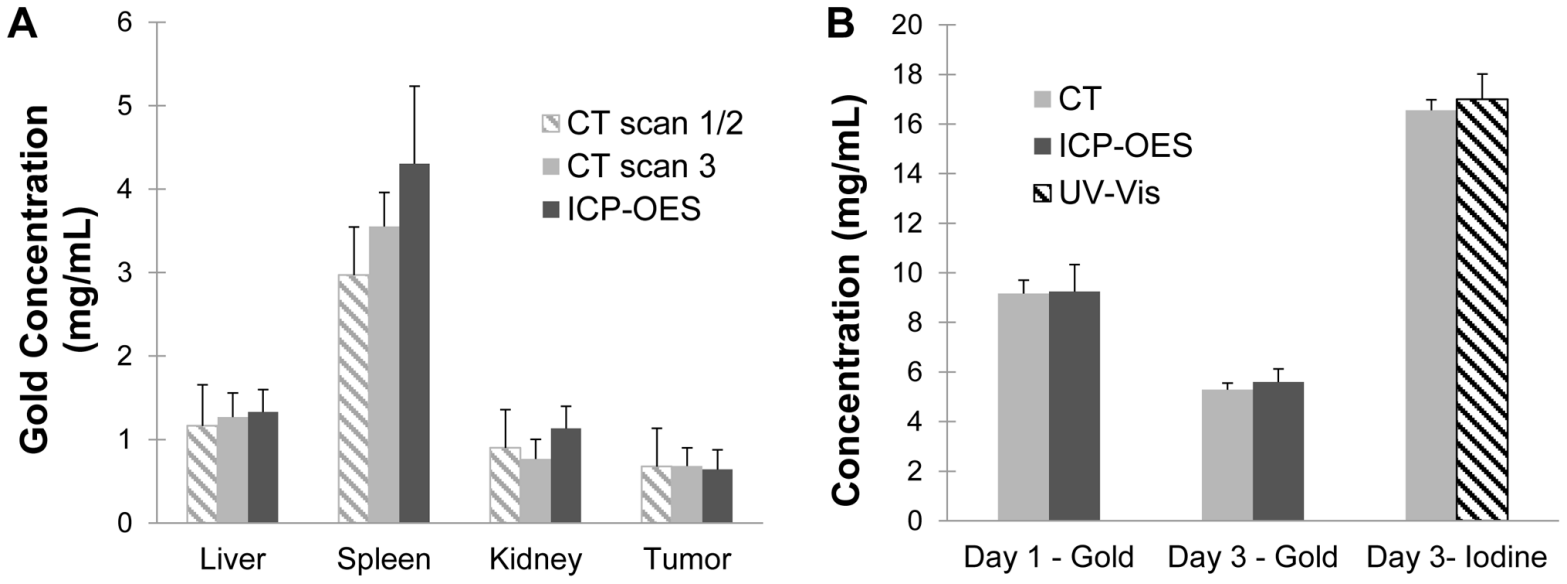
Comparison of CT-measured concentrations with validation methods. (A) Concentrations of accumulated gold in the tumors and other organs for the single-material method (based on CT scan 1 and 2), the two-material method (based only on CT scan 3) and ICP-OES. (B) Measured CT concentrations of gold in the blood on day 1 and day 3, iodine concentration in the blood on day 3, and corresponding measurements by ICP-OES and UV-Vis. None of the differences were found to be statistically significant (p<0.05).

Gold concentration within each tissue was calculated using both the segmented gold map from the DE decomposition and the ICP-OES results from the digested tissue and blood samples. Iodine concentrations in the blood were also determined using both the CT iodine map and absorbance spectra of blood samples. Figure 10 shows a comparison of the average CT-computed concentrations and the tissue sample concentrations for the five mice.

Figure 10a shows the calculated gold accumulation by CT (after subtracting the intravascular gold) for each organ versus the ICP-OES data. This figure shows the gold accumulation calculated by both CT methods. Data from CT scan 1 and 2 were used for the single contrast agent method, while data from CT scan 3 was used for the two contrast agent method. Both methods were compared with the ICP-OES results. The average gold accumulation in the tumors measured by CT using the dual energy method was 0.7±0.2 mg/mL. The spleen had the highest measured CT concentration of the other organs at 3.5±0.4 mg/mL, while the liver had 1.3±0.3 mg/mL and the kidneys had 0.8±0.2 mg/mL. In every case there was good agreement between the two CT methods as well as between the CT methods and the ICP-OES validation. 1-way ANOVA analysis of the three measurements for each organ was performed, and none of the differences were found to be statistically significant. Although it was not statistically significant, there was a small noticeable difference between the CT and ICP-OES data in the gold concentrations for the spleen and kidney.

Figure 10b shows the calculated gold and iodine concentrations in the blood by CT at each of the time points versus the ICP-OES or UV-Vis absorbance data. The average gold concentration in the blood immediately after injection on day 1 was 9.1 mg/mL, which fell to 5.3 mg/mL by day 3. The blood iodine concentration immediately after injection on day 3 was 16.6 mg/mL. In every case, there was exceptional agreement between blood concentrations calculated by CT and ICP-OES/UV-Vis. Paired t-tests of each comparison showed no statistically significant differences.

### Vascular Biomarkers Versus Tumor Size

**Figure 11:**
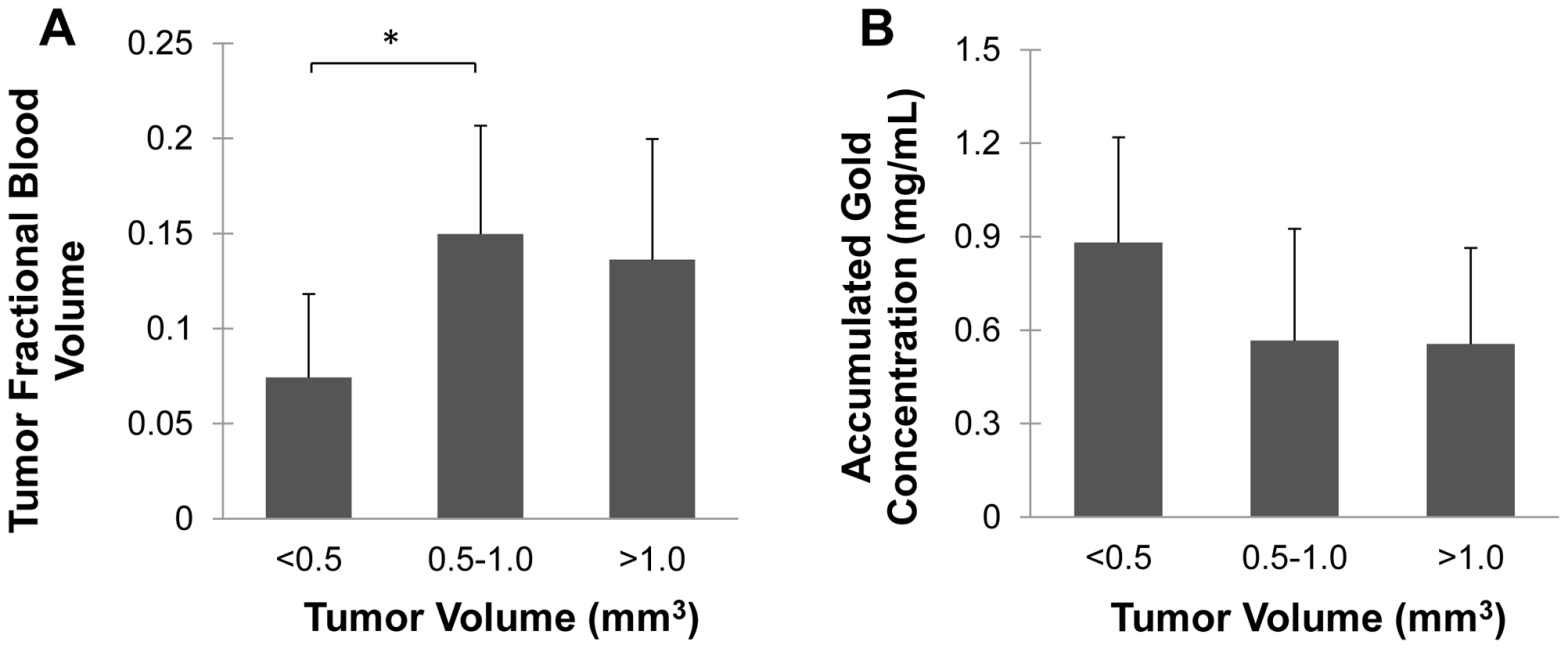
Tumor size and vascular biomarkers. Comparison of fractional blood volume (A) and accumulated gold concentration (B) for three different sizes of tumors. Statistical significance (p<0.05) is denoted by *.

Figure 11 shows a plot of FBV and accumulated gold concentration measurements versus tumor size. The ANOVA and post-hoc Tukey test results show a significant difference in FBV between the smallest (<0.5 mm^3^) tumors and the medium (0.5–1 mm^3^) tumors, with the medium tumors having a larger FBV. The large (>1 mm^2^) tumors also had a larger FBV than the smallest tumors, but the difference was not statistically significant (p = 0.08). The smallest tumors show a larger average gold concentration than the larger ones, but this difference does not reach statistical significance in the ANOVA (p = 0.10).

## Discussion

We have previously shown the ability to quantify vascular parameters with a single material and single x-ray energy for primary lung tumors [8]. We have also previously shown preliminary success in using our two-material, dual-energy method for quantifying tumor vascular parameters in soft-tissue sarcomas [14]. Extending this two-material DE micro-CT technique to lung tumor imaging presents considerable challenges. Primary lung tumors in mice are very difficult to image relative to other tumor types due to their small size (∼1-mm diameter in this study), cardiac and respiratory motion, and beam hardening artifacts. Micromotion during scanning can lead to very large differences in the reconstructed structure and enhancement of such small tumors. Even sub-voxel size motion can lead to volume averaging around the periphery of the tumor, giving the periphery an enhancement intermediate between tumor soft tissue and air-filled lung. While successful respiratory gating greatly reduces motion artifacts, micron-scale differences from projection to projection cannot be completely eliminated. In this study, some tumors looked different from one scan to the next (even on the same day). We presume this is due to small differences in positioning of the mice, differences in the depth of breathing between scans, and micromotion within a scan. All of these issues increased the difficulty of image analysis. Beam hardening due to nearby high-density structures (rib cage, vertebrae, contrast-filled heart and great vessels) causes additional artifacts due to changes in the x-ray spectrum as the rays pass through the animal. This can affect the 80 and 40 kVp scans differently, causing potentially significant inaccuracies in dual-energy decompositions. These issues make accurate quantitative dual-energy CT imaging of lung tumors one of the most difficult challenges in small animal imaging. The potential for errors in our calculations due to these challenges makes *ex vivo* validation of our results and comparison with our previous methods extremely important in assessing the adequacy of our method.

In this study, we performed three different DE micro-CT scans. The first two scans were done to replicate the single-material method used in our previous lung tumor study [8], but with a reduction of time (from 7 to 2 days) between the early and delayed imaging time points. This reduction in time is adequate for studies in mice with aggressive lung tumors. The third scan (after iodine injection) was performed to demonstrate our new method–using two materials and two x-ray energies to obtain the same vascular information when performing only a single CT scan. We injected gold 48 hours before our single scan to allow time for extravasation of the gold into the tumors and injected iodine immediately before our single scan to have a measure of FBV. From the dual-energy decomposition of our single scan, we could then calculate FBV based on the iodine map and vascular permeability based on the gold map.

Comparing the results of the first two CT scans (single-material method) with the results of the third CT scan (two-material method) showed that the two methods were in good agreement. FBV measurements made on day 1 and day 3 showed no statistically significant differences between the methods (see Figure 7). It is important to note that FBV was the same between the two methods, demonstrating reproducibility in measurements using two different long-circulating contrast agents. It also proves that the gold remaining in the bloodstream on day 3 did not interfere with the measurement of iodine for FBV calculation, even though there is a potential for increased beam hardening due to the presence of two contrast agents. Likewise, the accumulated tissue gold concentrations calculated for the two methods in both tumors and other organs were in agreement. There was a small (but not statistically significant) difference in the measured gold concentration in the spleen for the two methods, but no remarkable differences in any of the other tissues. Of all the organs that we measured, the spleen was the most prone to difficulties in the dual-energy decomposition due to its high vascularity and high gold uptake. The presence of high amounts of two contrast agents within the spleen may have led to some beam hardening, causing small inaccuracies in the dual-energy decomposition; however, this difference was small and not significant to the overall results of the study. Most importantly, the measurements for FBV and gold accumulation in the lung tumors agreed almost perfectly between the methods, showing that our two-material, single-scan method is just as good as (if not better) than our previous single-material, two-scan method for quantifying tumor vascular parameters.

Comparison of our two-material method with histological staining confirmed the accuracy of our FBV measurements. A comparison of microvascular density from CD31 immunostaining with FBV from CT showed a very strong correlation (R^2^ = 0.81), but not equivalence, between the two calculated parameters (see Figure 9). This is not surprising because, although they are clearly related, these two methods do not measure exactly the same parameters. Microvascular density measures the two-dimensional area of tissue sections that stains positive for an endothelial marker. It does not directly measure the vascular lumen or whether the vessels are perfused. FBV, on the other hand, is a three-dimensional measurement of perfused volume within the lumen of the vessels. Therefore, microvascular density is not inherently equal to the FBV, as we saw in our results. Although the two measurements are not equal, the strong correlation between them shows that differences in microvascular density as measured by CD31 staining can be accurately detected by *in vivo* CT FBV measurements.

Comparison of our two-material imaging method with ICP-OES tissue gold quantification and UV-Vis iodine quantification also confirmed the accuracy of our calculated gold concentrations (see Figure 10). Accumulated concentrations of gold in the tumors measured by CT matched almost perfectly with the ICP-OES results. Measurements of gold and iodine concentrations in the blood made by CT also matched the ICP-OES and UV-Vis absorbance results very closely. Accumulated concentration of gold in other organs, especially the spleen, showed a small (not statistically significant) difference between the calculated CT values and the measured ICP-OES values. This is most likely due to the assumptions made in CT and ICP-OES calculations. In the CT calculation for accumulated gold, intravascular gold was subtracted from the total gold concentration, leaving only the gold that has accumulated within the tumor tissue. Therefore, our accumulated gold concentrations assume that there is no intravascular gold remaining within the tissue. In our tissue processing, we drained the majority of the blood from the mouse before harvesting the organs and then floated the organs in PBS to remove additional blood; however, it is very unlikely that this method completely removed the blood from the organs. H&E sections showed some residual red blood cells within the larger vessels, showing that at least some blood remained. This explanation is further supported by the finding that the organ with the highest blood volume (spleen) showed the greatest discrepancy between CT and ICP-OES data. It is likely that the organs with the highest amount of blood before sacrificing the mouse still had the highest amount of blood (and therefore residual gold) after the blood was drained. The difference between the CT and ICP-OES for the tumors was smallest for the tumors, most likely because the tumors contained the least amount of blood to begin with and whatever blood remained was easily washed away in the PBS. Therefore, our assumption that the measured ICP-OES gold concentration should be equal to the extravasated gold concentration was most accurate for the tumors, and slightly less accurate for the larger, more vascular organs. Although small differences existed between the CT measurements and ICP-OES measurements, these differences were not statistically significant and not significant to the results of our tumor imaging. The overall agreement between the CT calculations and the ICP-OES results provided good validation of both our one-material and two-material methods for measuring vascular permeability.

Measurements of vascular permeability for both the one-material and two-material methods depended on the extravasation of gold nanoparticles. In this study, we used PEGylated 12 nm gold nanoparticles to estimate the vascular permeability of the tumors. This size was chosen to closely match the 15 nm commercially-produced AuroVist (Nanoprobes, Inc., Yaphank, NY) gold nanoparticles that had been used in our previous studies [14]. This choice allowed us to directly compare these results with the results from our previous two-material dual-energy study in sarcomas. This size of gold nanoparticle, however, may not be the best choice for maximizing specific gold accumulation in tumors. While we did see gold accumulation in the tumors, we also saw significant accumulation in other tissues, including the spleen, liver, kidneys, and normal lung tissue. Furthermore, a small accumulation was also present in muscle tissue, as confirmed by histology. This shows that our nanoparticles were small enough to extravasate in multiple non-tumor tissues. In choosing the ideal nanoparticle size, we aim to maximize both tumor specificity (less uptake in non-tumor tissues) and total tumor uptake and retention of our nanoparticles. The use of a larger size nanoparticle would likely reduce non-specific uptake in normal tissues and increase the total gold accumulation within tumors due to the EPR effect; however, larger nanoparticles may not be able to pass beyond the perivascular space in the tumors due to their size, which would prevent distribution of nanoparticles throughout the tumor. Previous studies have shown that maximal tumor uptake due to EPR occurs at sizes between 30 and 100 nm [38]. Other studies have shown that for targeted nanoparticles, the maximal specific uptake due to targeting is achieved at smaller sizes [39]. In addition, smaller nanoparticles are more likely to be able to spread throughout the tumor interstitial space, rather than being confined to the perivascular space. In order to optimize the choice of nanoparticle size in lung cancer imaging, our future studies will further explore the role of both nanoparticle size and specific receptor targeting on the uptake and distribution of nanoparticle in lung tumors.

The use of nanoparticles in tumor imaging has the potential for many additional benefits. Because nanoparticles are retained by tumors for a long period of time, they provide lasting contrast for the tumors when serial CT scans are needed, which would allow for tumor size monitoring over the course of cancer therapy. Measuring vascular permeability can also be used to predict which tumors are most likely to respond to nanoparticle chemotherapeutics [40]. Gold nanoparticle contrast agents could themselves also be used as combination agents for drug and gene delivery [41]–[43], photothermal therapy [44], or augmentation of radiotherapy [45].

The ideal result of this and subsequent studies would be the ability to distinguish different types of lung nodules and tumors by CT not only by their anatomical appearance, but also by other physiological parameters, including blood volume, vascular permeability, or even receptor expression. In this study we have used dual-energy CT to look at the differences between FBV and vascular permeability in different nodule sizes of lung adenocarcinomas. Although they are all the same tumor type, these tumors are at different levels of development. We have shown that there is a significant difference in fractional blood volume between small and medium-sized tumors. There is also a noticeable, but not statistically significant, difference in vascular permeability between small and large tumors. Our previous single-material study showed that there is a significant difference in vascular permeability between aggressive and benign lung tumors [8] and similar increased vascular permeability has been seen in other aggressive tumor models [40]. Other studies have shown that vascular permeability to nanoparticles changes as tumors transition from pre-malignant to malignant lesions [46], and that vascular permeability measurements correlate with levels of VEGF expression in tumors [47]. All of these results suggest the potential impact that functional CT imaging could have on lung tumor characterization. While one single parameter is not enough to fully characterize a tumor, multiple parameters used together could be very useful in discriminating tumor types. Additional studies are warranted to further compare these vascular measurements as well as other functional parameters in a variety of lung tumor and benign nodule types.

## Conclusions

We have shown that dual-energy micro-CT using gold and iodine nanoparticle contrast agents can accurately measure functional vascular parameters in a primary mouse model of lung cancer. Despite the challenges associated with imaging small lung tumors, *in vivo* dual-energy CT measurements of gold and iodine concentrations fully agree with the results of *ex vivo* studies. This dual-energy, two-material method is equivalent to our previous single energy, single material method, but allows for measurements of both fractional blood volume and vascular permeability with a single CT scan. This method has the potential for improving CT characterization of lung tumors, which could lead to better differentiation of different tumor types.

## Supporting Information

Figure S1 **Gold nanoparticle absorbance spectra.** Absorbance spectra of bare and PEGylated AuNPs, normalized to a peak absorbance of 1.0. The PEGylated AuNP peak is shifted ∼3 nm relative to the bare AuNP peak but is otherwise relatively unchanged.
(TIF)
