# Supplementary figures and images for: Dual-Energy Micro-CT Functional Imaging of Primary Lung Cancer in Mice Using Gold and Iodine Nanoparticle Contrast Agents: A Validation Study
Source: PLoS One. 2014 Feb 10;9(2):e88129. doi: 10.1371/journal.pone.0088129 (PMC3919743; doi:10.1371/journal.pone.0088129)

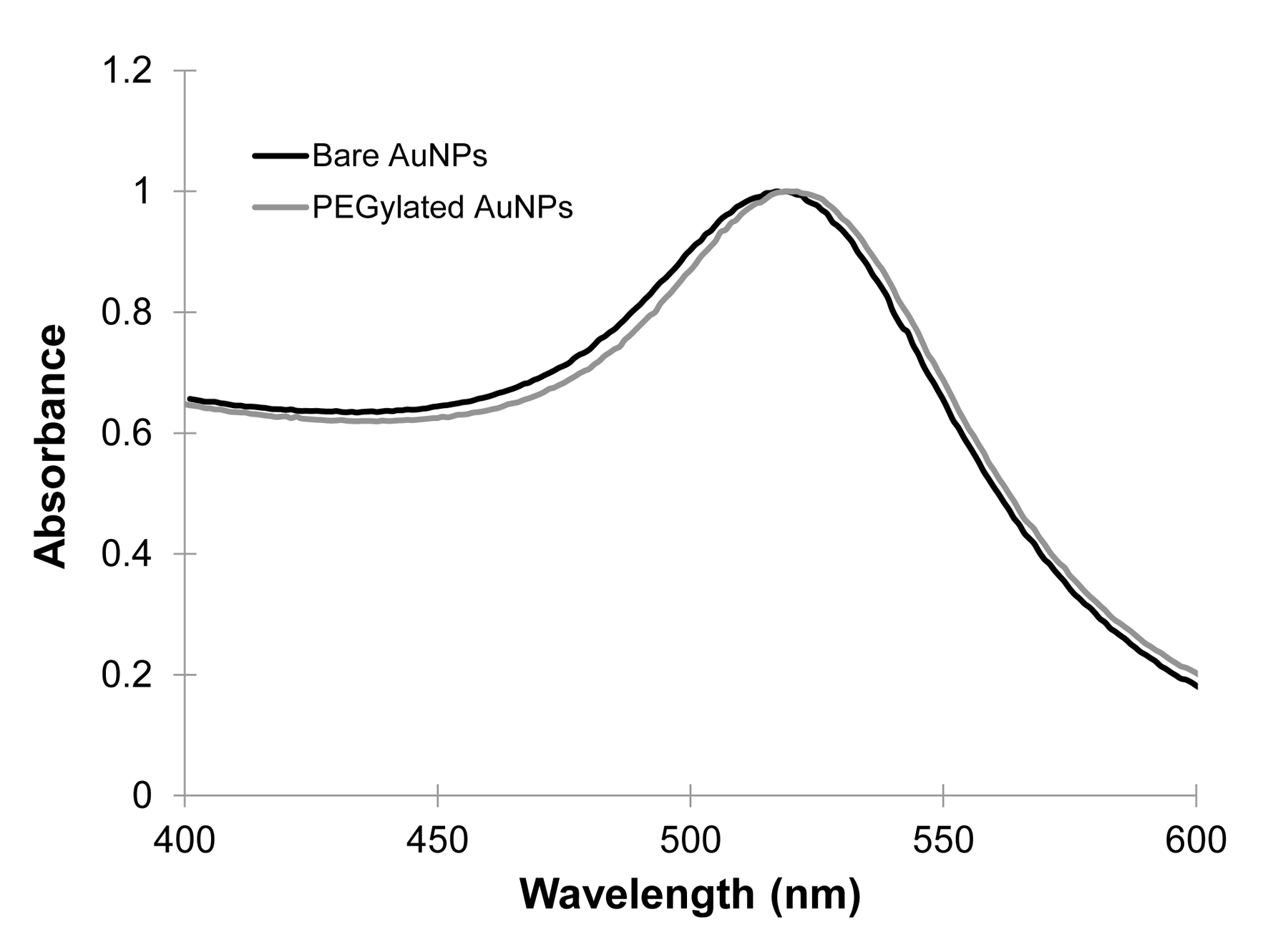

Supplement: Figure S1 — Gold nanoparticle absorbance spectra. Absorbance spectra of bare and PEGylated AuNPs, normalized to a peak absorbance of 1.0. The PEGylated AuNP peak is shifted ∼3 nm relative to the bare AuNP peak but is otherwise relatively unchanged. (TIF) [file pone.0088129.s001.tif]
